# A Case–Control Study of Lung Cancer Nested in a Cohort of European Asphalt Workers

**DOI:** 10.1289/ehp.0901800

**Published:** 2010-06-09

**Authors:** Ann Olsson, Hans Kromhout, Michela Agostini, Johnni Hansen, Christina Funch Lassen, Christoffer Johansen, Kristina Kjaerheim, Sverre Langård, Isabelle Stücker, Wolfgang Ahrens, Thomas Behrens, Marja-Liisa Lindbohm, Pirjo Heikkilä, Dick Heederik, Lützen Portengen, Judith Shaham, Gilles Ferro, Frank de Vocht, Igor Burstyn, Paolo Boffetta

**Affiliations:** 1 International Agency for Research on Cancer, Lyon, France; 2 Institute of Environmental Medicine, Karolinska Institutet, Stockholm, Sweden; 3 Institute for Risk Assessment Science, Utrecht University, Utrecht, the Netherlands; 4 Institute of Cancer Epidemiology, Danish Cancer Society, Copenhagen, Denmark; 5 The National Center for Cancer Rehabilitation Research, Institute of Public Health, Southern Danish University, Odense, Denmark; 6 Cancer Registry of Norway, Oslo, Norway; 7 Department of Occupational and Environmental Medicine, Oslo University Hospital, Ullevål, Oslo, Norway; 8 Institut National de la Santé et de la Recherche Médicale Unit U754, Villejuif, France; 9 Bremen Institute for Prevention Research and Social Medicine, Bremen, Germany; 10 Finnish Institute of Occupational Health, Helsinki, Finland; 11 School of Public Health, Tel Aviv University, Tel Aviv, Israel; 12 Occupational and Environmental Health Research Group, School of Translational Medicine, Faculty of Medical and Human Sciences, University of Manchester, Manchester, United Kingdom; 13 Community and Occupational Medicine Program, Department of Medicine, Faculty of Medicine and Dentistry, University of Alberta, Edmonton, Alberta, Canada; 14 The Tisch Cancer Institute, Mount Sinai School of Medicine, New York, New York, USA; 15 International Prevention Research Institute, Lyon, France

**Keywords:** bitumen, case–control studies, coal tar, dermal exposure, inhalation exposure, lung neoplasm, occupational exposure, polycyclic aromatic hydrocarbons

## Abstract

**Background:**

We conducted a nested case–control study in a cohort of European asphalt workers in which an increase in lung cancer risk has been reported among workers exposed to airborne bitumen fume, although potential bias and confounding were not fully addressed.

**Objective:**

We investigated the contribution of exposure to bitumen, other occupational agents, and tobacco smoking to the risk of lung cancer among asphalt workers.

**Methods:**

Cases were cohort members in Denmark, Finland, France, Germany, the Netherlands, Norway, and Israel who had died of lung cancer between 1980 and the end of follow-up (2002–2005). Controls were individually matched in a 3:1 ratio to cases on year of birth and country. We derived exposure estimates for bitumen fume and condensate, organic vapor, and polycyclic aromatic hydrocarbons, as well as for asbestos, crystalline silica, diesel motor exhaust, and coal tar. Odds ratios (ORs) were calculated for ever-exposure, duration, average exposure, and cumulative exposure after adjusting for tobacco smoking and exposure to coal tar.

**Results:**

A total of 433 cases and 1,253 controls were included in the analysis. The OR was 1.12 [95% confidence interval (CI), 0.84–1.49] for inhalation exposure to bitumen fume and 1.17 (95% CI, 0.88–1.56) for dermal exposure to bitumen condensate. No significant trend was observed between lung cancer risk and duration, average exposure, or cumulative exposure to bitumen fume or condensate.

**Conclusions:**

We found no consistent evidence of an association between indicators of either inhalation or dermal exposure to bitumen and lung cancer risk. A sizable proportion of the excess mortality from lung cancer relative to the general population observed in the earlier cohort phase is likely attributable to high tobacco consumption and possibly to coal tar exposure, whereas other occupational agents do not appear to play an important role.

Bitumen is the residual product from distillation of crude oil and is being used mainly as binder in asphalt mixes and in roofing applications (Asphalt Institute and Eurobitume 2008). Workers are primarily exposed to bitumen via inhalation or by skin contact (McClean et al. 2004a).

Bitumen fume and condensate contain a small fraction of polycyclic aromatic hydrocarbons (PAHs), of which benzo(*a*)pyrene is classified as a lung carcinogen by the International Agency for Research on Cancer (IARC) and others are suspected carcinogens (IARC 2010). Early epidemiologic studies of workers exposed to bitumen have suggested an increased risk of cancer, but the role of bitumen exposure in itself could not be disentangled from that of other occupational agents (in particular coal tar) and tobacco smoking (Partanen and Boffetta 1994; Schulte 2007). To investigate the risk of cancer among workers exposed to bitumen, a historical cohort study was conducted to investigate the mortality of European workers employed in road paving, asphalt mixing, waterproofing, and roofing (Boffetta et al. 1997, 2003a, 2003b). Road pavers represented the largest proportion of the study population. The workers were identified from companies in Denmark, Finland, France, Germany, Israel, the Netherlands, and Norway, and from a nationwide health surveillance program in Sweden. The mortality follow-up lasted between 1953 and 2000. The cohort study reported an increase in lung cancer mortality among workers exposed to bitumen fume overall and a relation between lung cancer mortality and increasing average exposure to bitumen fume, whereas a similar relation was not observed with increasing duration of exposure or cumulative exposure (Boffetta et al. 2001, 2003a, 2003b). Investigators in the Nordic countries also analyzed cancer incidence data; their results showed a small increase in lung cancer incidence (Kauppinen et al. 2003; Randem et al. 2003). However, the results of the mortality and the cancer incidence analyses could not contribute to a conclusion about the presence or absence of a causal link between exposure to bitumen fume and lung cancer because the assessment of bitumen exposure was rather crude, no information was available on employment in companies other than those included in the study, and very limited information was available for tobacco smoking (Burstyn et al. 2003). Subsequent sensitivity analyses, based on a Bayesian approach, suggested that neither latent confounding by smoking (de Vocht et al. 2009a) nor assumptions made in bitumen exposure assessment (de Vocht et al. 2009b) affected the conclusions of the cohort analyses with respect to lung cancer mortality.

A nested case–control study was therefore initiated to disentangle the contributions of bitumen, other agents occurring in the asphalt industry, other occupational exposures, and tobacco smoking to the increased risk of lung cancer observed among the asphalt workers in the cohort. This aim required the collection of more detailed information to better characterize exposure to bitumen and other agents in the asphalt industry and for information to be collected on other occupational exposure and smoking history. Although the assessment of exposure to bitumen was limited to inhalation (exposure to bitumen fume, organic vapors and PAHs) in the analysis of the whole cohort, in the nested case–control study, we also took into consideration the dermal route (exposure to bitumen condensate).

The objective of this study was to test whether the risk of lung cancer among asphalt workers is positively associated with exposure to bitumen, while adjusting for tobacco smoking and exposure to other known and suspected occupational lung carcinogens.

## Materials and Methods

**Figure 1:**
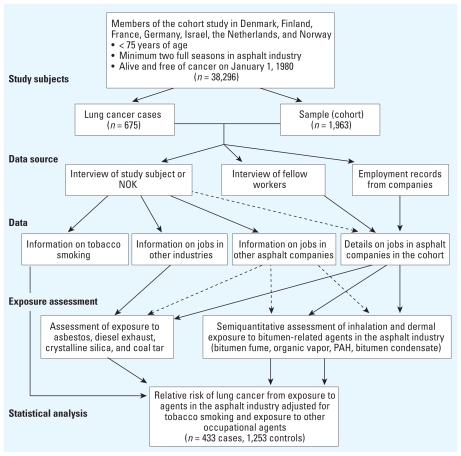
Outline of study methodology. NOK, next of kin. Dashed lines indicate minor contributions.

An outline of the methodology used in the study is presented in Figure 1. The study population (*n* = 38,296) consisted of male workers < 75 years of age who were included in the cohort study in Denmark, Finland, France, Germany, the Netherlands, Norway, and Israel; had been employed at least two full seasons in the companies included in the cohort (Boffetta et al. 2001); and were alive and free of cancer on 1 January 1980. The end of follow-up ranged from December 2002 in France to June 2005 in Finland. Ethical approval for conducting the study was obtained from the relevant ethics review committees. All participants provided informed consent to participate in the study.

Cases (*n* = 675) were members of the study population who died of lung cancer between 1980 and the end of follow-up, as well as incident cases identified through cancer registries during the same periods in Denmark, Finland, Norway, and Israel. For each case, a set of controls was selected randomly among members of the study population if they fulfilled the matching criteria (birth year ± 3 years, country) and were free of respiratory and ill-defined cancer [*International Classification of Diseases, 9th Revision* (ICD-9), neoplasm codes 160–165 and 195–199); World Health Organization (WHO) 1975] at the age of diagnosis (among incident cases) or the death of the case. A list was prepared of eight eligible controls for each case; we contacted these individuals in the order they appeared on the list. If an address or telephone number could not be found, we went on to the next person on the list until three workers were located.

We sent an invitation letter to the worker (if alive) or to a next of kin (NOK) as soon as an address was identified. The letter was followed up by telephone calls. Some cohort members were selected as potential controls for more than one case, as it is in incidence-density sampling of controls within cohorts (Lubin and Gail 1984). Thus, if they were interviewed, they were treated as multiple individuals in the statistical analysis.

Telephone interviews based on a structured questionnaire designed for this study were conducted with the study subjects or their NOK to obtain information on demographics, smoking history, and lifetime work history within and outside the asphalt industry. A complete interview lasted, on average, about 45 min.

Each country in the study determined the best procedures for tracing and contacting the subjects. In general, the addresses of study subjects and NOK were obtained from company records, population registries, pension or insurance files, or telephone directories. The last spouse was the preferred NOK to be interviewed when the index person was not available. If the last spouse was not available, a previous spouse, a descendant, a sibling, another relative, a neighbor, or a friend was selected in decreasing order of preference. If an interviewer considered an interview to be of low quality, a second person and sometimes a third were interviewed. The most informative interview according to the interviewer was selected for the analysis.

Questionnaires were translated into the local language in each of the centers by the study team with assistance from industry representatives. Thereafter, a native speaker at IARC reviewed the translation by comparing the national version with the original English version. The general occupational history was coded according to the International Standard Industrial Classification (United Nations 1971) and the NACE Rev 1.1 Classification of Economic Activities (European Commission 2002) for industries and the International Standard Classification of Occupations (International Labour Organization 1968) for job titles.

We collected detailed information on jobs held within the asphalt industry from living subjects and fellow workers who had worked alongside the study subjects. Information collected from the companies during the cohort phase served as a starting point, which the interviewed person could corroborate, refute, or amend. Fellow workers were identified through the occupational history collected in the main interview, through industry representatives, through matching of the cohort records, and by asking the NOK.

We obtained semiquantitative exposure estimates for bitumen fume, organic vapors, and 4- to 6-ring PAHs for 85 specific jobs in the asphalt, building, and ground construction industry from the Asphalt Workers Exposure database (Burstyn et al. 2000; Burstyn and Kromhout 2000). These exposure estimates were included in algorithms together with other parameters to calculate individual exposure levels (Agostini et al. in press). For example, the work-time parameter was based on the median length of the paving season, work week, and workday as reported for each job and time period by the companies. This number was divided by 480 (12 months × 5 days × 8 hr) to arrive at the work-time modifier included in the algorithms. A multiplier for the use of coal tar was applied in the algorithm for estimating exposure to PAH (Burstyn et al. 2000). Information on coal tar use and oil gravel paving came from the company questionnaires that were collected during the cohort phase as a primary source (Burstyn et al. 2003). If this information was lacking, we used information from interviews of fellow workers or from country-specific local industry experts.

Estimates for dermal exposure to bitumen condensate were based on a relative ranking of the 85 jobs identified within the asphalt, building, and ground construction industries. The information came from structured semiquantitative dermal exposure assessment (DREAM) observations of paving and mastic crews in Germany, Denmark, France, and the Netherlands (Van-Wendel-de-Joode et al. 2003) and dermal exposure measurement surveys (Cirla et al. 2005; Jongeneelen et al. 1988; McClean et al. 2004a, 2004b, 2007; Väänänen et al. 2005, 2006). For jobs without DREAM observations or measurements, two industrial hygienists independently estimated exposure. The consensus score was used in the analysis. We applied a similar time trend for bitumen condensate as for inhalation exposure to bitumen fume. Assessment for dermal coal tar exposure could not be performed because of the absence of relevant data. Dermal exposure estimates were, like the inhalation exposure estimates, adjusted for actual time worked within each calendar period. In addition, we applied a hygienic behavior multiplier to the algorithms to take into account clothing patterns, use of personal protective devices (e.g., gloves) and hygienic behavior (e.g., showering, cleaning hands with solvents or fuels). Similarly, we estimated the hygienic behavior modifier at company, job class, and calendar-period, based on reported information coming from living subjects and fellow workers. Optimal hygienic behavior (wearing a coverall, no short sleeves, no shorts, not working with bare trunk, wearing gloves, showering or bathing directly after work, and cleaning hands with water and soap) resulted in a low score leading to a low multiplier (0.1), whereas seven poor hygienic behavior scores resulted in no adjustment, because the hygienic behavior multiplier would be 1.

When we did not have work time and hygienic behavior information reported for a certain period, we had to extrapolate data. In cases where we had estimates for an earlier or later period, we used the nearest (timewise) estimate. Otherwise we took the median of the values for other companies in the same country in the same time period for the same job class.

We assumed asbestos, coal tar, crystalline silica, and diesel motor exhaust to be the exposures with the highest expected prevalence and potential for confounding the association with bitumen-related agents. Exposure to these agents was estimated by applying two exposure matrices; one for inside and one for outside the asphalt, building, and ground construction industry. Two industrial hygienists independently gave scores (0, 1, and 2 referring to “no”, “low”, and “high,” respectively), and the consensus score was kept. A similar approach was taken for jobs outside the asphalt or construction industry coded by the International Standard Classification of Occupations (International Labour Organization 1968) and the International Standard Industry Classification (United Nations 1971). A total of 1,297 job-industry combinations were evaluated in this way. We made no time period-specific adjustments, except for in the asphalt industry, where exposure to asbestos or coal tar only was assigned when an individual worked during the time when these agents were still being used. The end-of-use years for coal tar were mainly derived from the information provided by the companies in the company questionnaires that were collected during the cohort phase. A similar method was adopted for asbestos. We used the information from the fellow-worker interviews when this information was lacking in the original company questionnaires.

We linked the two job exposure matrices to each individual and squared the intensity scores to take into account the lognormal nature of exposure concentrations, and then multiplied by duration to get an indicator expressed as cumulative exposure-years. Because the same scale for intensity was used in both matrices, we were able to sum the exposures to these agents across the full job history for each individual.

In preliminary analyses, we compared the results of conditional and unconditional logistic regression models and found no differences. Therefore, in the analysis presented here, unconditional logistic regression models were fitted to calculate odds ratios (ORs) with 95% confidence intervals (CIs) of lung cancer for each agent, adjusted for matching set, age group (< 60, 60–64, 65–69, and 70–74 years), country, and cumulative tobacco smoking (< 10, 10–19, 20–39, and ≥ 40 pack-years) (Breslow and Day 1980). Because of the correlation observed between coal tar and bitumen exposures, the ORs for exposure to bitumen fume and bitumen condensate were also adjusted for having ever been exposed to coal tar. We used SAS PROC LOGISTIC (version 9.1; SAS Institute Inc., Cary, NC, USA) to perform the statistical analyses.

For each agent, we assessed four dimensions of exposure: ever-exposure, duration of exposure, cumulative exposure, and average exposure. In preliminary analyses, we also considered 15-year lagged cumulative exposure and 15-year lagged average exposure, in which exposure in the past 15 years before diagnosis was disregarded. Because these variables did not provide additional insight in the results compared with the respective unlagged variables, they were not considered in any further analyses.

For continuous variables, exposed subjects were categorized into quartiles, with cutoff points based on the distribution among controls and unexposed subjects forming the reference category.

Tests for linear trends (across all subjects and across exposed subjects only) were calculated by comparing the log likelihood ratio of a model without the variable of interest with that of a model including the variable on a continuous scale, with values corresponding to the mid-interval of exposure score values in each category. Heterogeneity across countries was tested comparing the log likelihood ratio of a model with an interaction term between the variable of interest and country to that of a model without it.

Missing data on tobacco smoking were not imputed. For quantitative tobacco smoking variables, this resulted in several subjects being classified in a group with unknown exposure level, which was analyzed separately.

Furthermore, ORs and 95% CIs of participation in the case–control study were calculated for the exposure variables available in the cohort phase of the study, after adjusting for age, country, and case–control status. The dependent variable in the logistic regression models was participation in the case–control study. The aim of this analysis was to assess whether participation in the study was associated with exposure as assessed during the cohort phase.

The possible confounding effect exerted by tobacco smoking in the analysis of the cohort based on national mortality rates was assessed by calculating country-specific confounding odds ratio (COR) according to the following formula (Axelson and Steenland 1988):


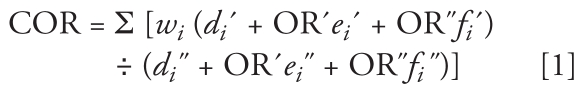


where, in the two age groups (45–64 and ≥ 65, subscript i), *d*′, *e*′, and *f*′ are the proportions of nonsmokers, ex-smokers, and current smokers among living controls belonging to the same birth cohorts as the participants of the surveys, *d*″, *e*″, and *f*″ are the corresponding proportions in national surveys, and *w* are the weights (based on the distribution of person-years in the cohort in the two age groups). The ORs of lung cancer for ex-smokers (OR′) and current smokers (OR″) were set to 4 and 9, respectively (Gandini et al. 2008).

National survey data on prevalence of smoking were obtained from the Closing the Gap project (Zatonski 2008), with the exception of Norway (Lindbak RL, personal communication) and Israel (Israel Center for Disease Control 2008).

## Results

A total of 436 cases were identified and agreed to be interviewed; the overall response rate was 65%, with an intercountry range of 37% (the Netherlands) to 86% (Denmark). Three cases were excluded because no controls could be identified for them, leaving 433 cases in the analysis (Table 1). We attempted to contact 1,963 of the 5,052 eligible controls; of these, 1,692 were successfully contacted and 1,131 were interviewed; the overall response rate was 58% (21% in the Netherlands, 74% in Israel). A total of 184 controls were matched to more than one case. Thus, 1,253 controls were included in the analysis (Table 1). Of those interviewed, 2% of cases (*n* = 9) and 66% of controls (*n* = 824) were interviewed in person. Spouses (54% of cases, 44% of controls) and children (36% of cases, 49% of controls) were the most commonly interviewed NOK. Nonrelative NOK was the source of information for 4% of cases and 1% of controls.

As a result of the matching, the age distribution of cases and controls was very close. Only 8 cases (1.8%) were never smokers compared with 16.4% of controls (Table 1). The ORs for ex-smoking and current smoking were 6.12 (95% CI, 2.92–12.81) and 15.98 (95% CI, 7.72–33.06), respectively. Strong exposure–response relationships were observed for cumulative pack-years of smoking, duration of smoking, average daily consumption, and time since quitting smoking [see Supplemental Material, Table S1 (doi:10.1289/ehp.0901800)]. There was no correlation between tobacco smoking and cumulative or average exposure to bitumen-related agents, with the exception of a very weak but statistically significant correlation with average exposure to bitumen fume (correlation coefficient 0.07, *p* = 0.003).

The OR for ever-exposure to bitumen fume was 1.12 (95% CI, 0.84–1.49) (Table 2), and there was no relation between lung cancer risk and duration of exposure, cumulative exposure, or average exposure (Table 3). Results for exposure to organic vapor and PAH were similar to those for exposure to bitumen fume (Table 2); results by indicators of semiquantitative exposure are available in Supplemental Material, Table S2 (doi:10.1289/ehp.0901800).

The OR for ever-exposure to bitumen condensate was 1.17 (95% CI, 0.88–1.56) (Table 2). There was no association with duration of exposure, cumulative exposure, or average exposure to this agent: the OR in the category at highest average exposure to bitumen condensate was 1.23 (95% CI, 0.81–1.88; *p*-value of test for linear trend 0.26) (Table 4).

The analysis of exposure to asbestos, crystalline silica, and diesel motor exhaust did not reveal any association with lung cancer risk [see Supplemental Material, Table S3 (doi:10.1289/ehp.0901800)]. On the other hand, an association was suggested between lung cancer risk and cumulative exposure to coal tar and, to a lesser extent, duration of exposure (Table 5).

For 277 cases (66.7%), information was available on histological type: 99 (35.7%) were squamous cell carcinomas, 73 (26.4%) were adenocarcinomas, 50 (18.1%) were small-cell carcinomas, and the remaining 55 cases (19.9%) had either other or mixed histology. Although results for squamous cell carcinoma tended to be slightly more positive than those for adenocarcinoma and small-cell carcinoma [in particular, the OR of squamous cell carcinoma in the highest category of average exposure to bitumen condensate was 1.93 (95% CI, 0.85–4.39)], none of the differences were statistically significant (e.g., *p*-value of test of heterogeneity for ever-exposure to bitumen condensate was 0.8).

The results of models including adjusting for tobacco smoking and coal tar exposure (OR2 in Tables 3 and 4) were comparable with results without such adjustment (OR1 in Tables 3 and 4). This finding suggests that these factors, as measured in this study, exerted little confounding effect on the association between bitumen exposure and lung cancer risk. Similarly, the inclusion of terms for exposure to other occupational agents, one by one and all together, suggested that none of these agents exerted a confounding effect on the association between lung cancer risk and occupational exposures to bitumen fumes [see Supplemental Material, Table S4 (doi:10.1289/ehp.0901800)].

**Figure 2:**
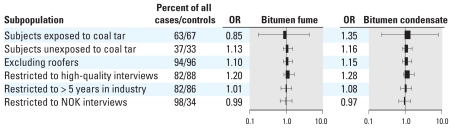
Results of selected sensitivity analyses. Lung cancer OR and 95% CI for ever-exposure to bitumen fume (inhalation) and bitumen condensate (dermal).

To better explore the possible confounding effect of coal tar exposure, we stratified the analysis of bitumen fume and bitumen condensate by coal tar exposure. The results for ever-exposure are reported in Figure 2 and show no heterogeneity. Some heterogeneity was suggested when semiquantitative bitumen exposure variables were analyzed; for example, OR was 1.02 for high average exposure to bitumen condensate among subjects unexposed to coal tar and 1.49 among subjects exposed to coal tar, but none of these differences were statistically significant [see Supplemental Material, Table S5 (doi:10.1289/ehp.0901800)]. Exclusion of workers who were ever employed as roofers (28 cases, 54 controls) (Figure 2) and workers who were ever employed in mastic asphalt paving (4 cases, 8 controls; not shown in detail) had no material impact on risk estimates.

To further assess the robustness of the results, we excluded interviews of medium or low quality, by restricting the analysis to subjects with 5 or more years of employment in the asphalt industry, and by restricting the analysis to NOK interviews for deceased cases and controls (Figure 2). The unexposed subjects were the reference category in each of the analyses. These exclusions did not provide evidence of selection or information bias, although restriction of the analysis to high-quality interviews resulted in slightly higher risk estimates.

We assessed the contribution of individual countries to the overall result by excluding one country at a time. The ORs for ever-exposure to bitumen fume ranged from 1.06 (exclusion of France, 95% CI, 0.78–1.45) to 1.21 (exclusion of Norway, 95% CI, 0.87–1.68). The corresponding ORs for ever-exposure to bitumen condensate ranged from 1.13 (exclusion of Finland, 95% CI, 0.83–1.53) to 1.26 (exclusion of Norway, 95% CI, 0.90–1.76); see Supplemental Material, Table S6 (doi:10.1289/ehp.0901800).

We studied what characteristics were associated with participation in the current study. Case or control status and having been employed as a paver were not associated, whereas long duration of employment and long duration of exposure to bitumen (as estimated in the cohort phase of the study) were associated with participation. There was no association between cumulative semiquantitative exposure to bitumen as assessed in the cohort study and participation in the case–control study (not shown).

The proportion of smokers was higher among controls than among persons in the general population in almost all the countries. The estimated confounding ORs were 1.07 in the Netherlands, 1.12 in Israel, 1.14 in France, 1.24 in Norway, 1.25 in Germany, and 1.28 in Denmark and Finland.

## Discussion

This case–control study of lung cancer nested in the cohort of European asphalt workers was designed to explore the role of bitumen exposure, other occupational exposures, and tobacco smoking in determining the increased mortality from lung cancer observed among pavers in the cohort (Boffetta et al. 2001, 2003a, 2003b). The inclusion of the assessment of dermal exposure to bitumen condensate is an innovative methodological feature of the study.

The main results of the study are (*a*) no significant association between indicators of inhalation and dermal exposure to bitumen and lung cancer, (*b*) the lack of an effect of other known or suspected occupational lung carcinogens in the asphalt industry or in other jobs, with the possible exception of exposure to coal tar, and (*c*) a higher prevalence of tobacco smoking in the study population compared with national surveys, which might have biased the results of the cohort study away from the null.

Caution should be used in comparing the results of the cohort and the nested case–control study for the following reason: not all cases identified in the cohort analysis were included in the case–control study because of exclusion of

- One country (Sweden)
- Workers with less than two seasons of employment
- Subjects who died before 1980
- Workers employed in the job classes representing administrative and office work
- Subjects who were not reached or did not want to participate in the case–control study.

In addition, the case–control analysis included additional cases of lung cancer identified after the end of the follow-up in the cohort analysis; 217 (50%) of the current cases were included after the cohort study was completed. Occupational exposure levels of bitumen fume have decreased during the last decades (Burstyn et al. 2003); therefore, cases from earlier time periods included only in the cohort study might have been exposed, on average, to higher levels of bitumen fume and other agents compared with the cases included in the nested case–control study. The results of this study, therefore, reflect the effects of exposure circumstances prevalent in recent decades.

This study has several strengths. The detailed assessment of exposure to bitumen by both inhalation and dermal routes and the assessment of exposure to other agents both within and outside the asphalt industry led to improvement in the quality of information on work histories compared with the cohort phase of the study and relative to other studies of cancer risk among bitumen-exposed workers (Partanen and Boffetta 1994). In particular, more detailed job histories in the asphalt industry were collected; 95.8% of work-history years could be coded at the level of specific jobs or tasks within an occupation (Agostini et al. in press). Exposure intensity models developed and validated for the cohort study (Burstyn et al. 2003) were used in modeling exposures in the case–control study; additional models of dermal exposure were developed on the basis of newly available measurements and observations. The case–control study attained satisfactory response rates among both cases and controls (Kjaerheim et al. 2002). The results on the carcinogenic effect of tobacco smoking are consistent with the expected relationship (Gandini et al. 2008) and allowed fair adjustment for smoking. The consistency of results among countries and the robustness with respect to indicators of quality of data are further arguments in favor of the credibility of our results.

The main limitations of the study are the lack of some of the elements needed for exposure assessment at the individual level and the semiquantitative nature of exposure estimation. Contrary to initial plans, the data collected from fellow workers were too sparse to allow the modulation of job-history–based exposure estimates at the individual level, for example, related to personal hygiene. Thus, we used this information by company, job, and time period, because living subjects providing this information were predominantly controls, whereas the corresponding information for deceased cases was obtained from NOK and fellow workers. Using this information at the individual level could have introduced bias.

Other potential limitations of the study, such as the higher proportion of controls with in-person interviews compared with cases, the variable number of controls available for cases, and the low quality of a subset of interviews, were addressed in sensitivity analyses and did not appear to have affected the results. The low response rate in some of the countries (e.g., among controls in the Netherlands) is an additional limitation; in sensitivity analyses, however, individual countries did not appear to influence the results.

Exposure misclassification due to inaccuracy in individual exposure estimates might have occurred; if exposure misclassification was nondifferential, it most likely would have resulted in attenuation of risk estimates.

The assessment of occupational exposures to agents other than those related to bitumen was rather crude, which is reflected by lack of effect for most of them. This can be explained by misclassification, but can also be attributed to the narrow range of exposure experienced by asphalt and construction workers and low levels as a result of declining exposure levels in the work sites (Burstyn et al. 2003). In addition, the power to detect small effects is limited when the prevalence of co-exposures are as frequent as in this study, for example, the prevalence of diesel motor exhaust exposure among the controls was 95%, resulting in a power ~ 25% to detect an OR of 1.5.

The confounding OR by smoking ranged from 1.07 to 1.28 among the participating countries. Therefore, the comparison of the distribution of smoking among living controls and national surveys may suggest that a sizable proportion of the excess mortality from lung cancer observed when the cohort of asphalt workers was compared with national mortality rates, can be explained by the higher prevalence of smoking among cohort members. However, the comparison of smoking prevalence between the case–control study and the national surveys is limited by several factors, including possible lack of correspondence of definition of smokers in the surveys and in the nested case–control study, a healthy survivor effect (linked to lower smoking prevalence) among living controls, and the limited response rate among controls. The confounding ORs should therefore be interpreted only in qualitative terms.

Tobacco smoking was not strongly correlated with cumulative or average exposure of the agents studied, but entailed some confounding effect and was controlled for in the analysis.

Exposure to other lung carcinogens outside the occupational environment, such as indoor radon, would exert a confounding effect only if these factors are correlated with the agents under study (e.g., average exposure to bitumen fume or bitumen condensate), which is not very plausible.

Sixteen comparisons were performed in the main analysis (four dimensions of exposure to four bitumen-related agents), and a Bonferroni correction would set the *p*-value of statistical significance at 0.003, a level that was not approached by any result. Although the Bonferroni correction might be too conservative because the exposure variables were not independent, chance remains a plausible explanation of the results for the slight increase in OR for some of the exposure variables.

The results of this study are consistent with the recent evaluation of IARC (2010) of an increased risk of lung cancer among pavers and roofers exposed to coal tar. They also contribute to the interpretation of results of previous cohort studies of workers exposed to bitumen with no or limited exposure to coal tar (reviewed by Armstrong et al. 2004, and Partanen and Boffetta 1994).

## Conclusions

Two main conclusions can be drawn from the present study. First, a sizable proportion of the excess mortality from lung cancer relative to the general population observed during the cohort phase of the study is likely attributable to the high consumption of tobacco experienced by these workers and possibly to coal tar exposure, whereas other occupational agents do not appear to play important roles. Second, we found no consistent evidence of an association between indicators of inhalation or dermal exposure to bitumen and lung cancer risk. However, our study underscores the importance of the current trend in reducing inhalation and dermal exposure to bitumen in the workplace, as our study may have failed to detect weak yet important exposure–response associations.

## Figures and Tables

**Table 1:** Description of the study population.

Variable	Cases (*n* = 433) (*n*) (%)	Controls (*n* = 1,253) (*n*) (%)
Country		
Denmark	139 (32.1)	393 (31.4)
Finland	37 (8.5)	111 (8.9)
France	73 (16.9)	218 (17.4)
Germany	63 (14.5)	198 (15.8)
Israel	18 (4.2)	47 (3.8)
Netherlands	21 (4.8)	58 (4.6)
Norway	82 (18.9)	228 (18.2)

Age (years)		
< 60	131 (30.3)	387 (30.9)
60–64	96 (22.2)	277 (22.1)
65–69	108 (24.9)	324 (25.9)
70–75	98 (22.6)	265 (21.1)

Tobacco smoking		
Never smoker	8 (1.8)	206 (16.4)
< 10 pack-years	22 (5.1)	153 (12.2)
10–19 pack-years	44 (10.2)	153 (12.2)
20–39 pack-years	91 (21.0)	292 (23.3)
40–186 pack-years	169 (39.0)	305 (24.3)
Unknown pack-years	99 (22.9)	144 (11.5)

Occupational exposure to crystalline silica		
Never	108 (24.9)	291 (23.2)
Ever	325 (75.1)	962 (76.8)

Occupational exposure to diesel motor exhaust		
Never	39 (9.0)	62 (4.9)
Ever	394 (91.0)	1,191 (95.1)

Occupational exposure to asbestos		
Never	126 (29.1)	331 (26.4)
Ever	307 (70.9)	922 (73.6)

Occupational exposure to coal tar		
Never	274 (63.3)	843 (67.3)
Ever	159 (36.7)	410 (32.7)

**Table 2:** Ever-exposure to bitumen-related agents and lung cancer risk.

Exposure	Category	Cases *n* (%)	Controls *n* (%)	OR^a^ (95% CI)
Bitumen fume^b^, ^c^	Never	130 (30.0)	412 (32.9)	1.00
	Ever	303 (70.0)	841 (67.1)	1.12 (0.84–1.49)
Organic vapor^b^	Never	130 (30.0)	412 (32.9)	1.00
	Ever	303 (70.0)	841 (67.1)	1.20 (0.93–1.55)
PAH^b^	Never	56 (12.9)	191 (15.2)	1.00
	Ever	377 (87.1)	1,062 (84.8)	1.20 (0.85–1.69)
Bitumen condensate^c^, ^d^	Never	124 (28.6)	403 (32.2)	1.00
	Ever	309 (71.4)	850 (67.8)	1.17 (0.88–1.56)

a Adjusted for set, country, age, tobacco pack-years.

b Inhalation exposure.

c Also adjusted for coal tar exposure.

d Dermal exposure.

**Table 3:** Inhalation exposure to bitumen fume and lung cancer risk.

Exposure category	Cases *n* (%)	Controls *n* (%)	OR1^a^ (95% CI)	OR2^b^ (95% CI)
Never^c^	130 (30.0)	412 (32.9)	1.00 (Reference)	1.00 (Reference)

Duration of exposure (years)				
0.33–7.99	85 (19.6)	208 (16.6)	1.32 (0.95–1.83)	1.19 (0.84–1.69)
8.00–15.49	82 (18.9)	208 (16.6)	1.26 (0.91–1.76)	1.26 (0.87–1.83)
15.50–25.99	81 (18.7)	205 (16.4)	1.26 (0.91–1.76)	1.23 (0.84–1.79)
26.00–54.00	55 (12.7)	220 (17.6)	0.78 (0.54–1.12)	0.74 (0.49–1.11)
Test for linear trend, *p*-value			0.51	0.37

Cumulative bitumen fume exposure (unit-years)				
0.18–9.55	88 (20.3)	211 (16.8)	1.34 (0.97–1.85)	1.31 (0.93–1.85)
9.56–28.17	73 (16.9)	210 (16.8)	1.09 (0.78–1.54)	0.99 (0.68–1.45)
28.18–68.00	82 (18.9)	208 (16.6)	1.24 (0.88–1.73)	1.16 (0.78–1.72)
68.01–620.48	60 (13.9)	212 (16.9)	0.87 (0.60–1.25)	0.77 (0.50–1.19)
Test for linear trend, *p*-value			0.76	0.39

Average exposure to bitumen fume (units)				
0.08–0.97	78 (18.0)	209 (16.7)	1.18 (0.84–1.64)	1.20 (0.84–1.71)
0.98–2.20	75 (17.3)	211 (16.8)	1.13 (0.81–1.59)	1.15 (0.78–1.70)
2.21–3.61	65 (15.0)	209 (16.7)	0.98 (0.69–1.40)	0.90 (0.60–1.34)
3.62–16.67	85 (19.6)	212 (16.9)	1.27 (0.91–1.78)	1.16 (0.78–1.73)
Test for linear trend, *p*-value			0.33	0.80

a Adjusted for set, country, and age.

b Adjusted for set, country, age, tobacco pack-years, and coal tar exposure.

c Referent group for all analyses shown in the table.

**Table 4:** Dermal exposure to bitumen condensate and lung cancer risk.

Exposure category	Cases *n* (%)	Controls *n* (%)	OR1^a^ (95% CI)	OR2^b^ (95% CI)
Never^c^	124 (28.6)	403 (32.2)	1.00 (Reference)	1.00 (Reference)

Duration of exposure (years)				
0.33–7.99	85 (19.6)	211 (16.8)	1.33 (0.96–1.86)	1.22 (0.86–1.74)
8.00–15.49	84 (19.4)	209 (16.7)	1.32 (0.95–1.85)	1.34 (0.93–1.94)
15.50–26.49	89 (20.6)	218 (17.4)	1.34 (0.97–1.86)	1.35 (0.93–1.96)
26.50–54.00	51 (11.8)	212 (16.9)	0.77 (0.53–1.12)	0.72 (0.47–1.10)
Test for linear trend, *p*-value			0.67	0.50

Cumulative bitumen condensate exposure (unit-years)				
0.59–61.54	79 (18.2)	213 (17.0)	1.21 (0.87–1.68)	1.21 (0.85–1.72)
61.55–185.25	81 (18.7)	212 (16.9)	1.24 (0.89–1.74)	1.22 (0.84–1.76)
185.26–407.07	66 (15.2)	213 (17.0)	1.01 (0.71–1.43)	0.99 (0.66–1.49)
407.08–4003.76	83 (19.2)	212 (16.9)	1.28 (0.91–1.80)	1.21 (0.79–1.84)
Test for linear trend, *p*-value			0.32	0.58

Average exposure to bitumen condensate (units)				
0.29–6.62	70 (16.2)	223 (17.0)	1.06 (0.76–1.49)	1.10 (0.77–1.57)
6.63–13.44	74 (17.1)	212 (16.9)	1.15 (0.81–1.62)	1.21 (0.83–1.76)
13.45–23.06	80 (18.5)	212 (16.9)	1.25 (0.89–1.75)	1.25 (0.84–1.87)
23.07–94.11	85 (19.6)	213 (17.0)	1.33 (0.95–1.87)	1.23 (0.81–1.88)
Test for linear trend, *p*-value			0.07	0.26

a Adjusted for set, country, and age.

b Adjusted for set, country, age, tobacco pack-years, and coal tar exposure.

c Referent group for all analyses shown in the table.

**Table 5:** Exposure to coal tar and lung cancer risk.

Exposure category	Cases (*n*)	Controls (*n*)	OR^a^ (95% CI)
Never^b^	274	843	1.00 (Reference)

Duration of exposure (years)			
0.33–3.99	38	85	1.45 (0.94–2.25)
4.00–8.49	31	114	0.83 (0.53–1.30)
8.50–13.49	44	107	1.40 (0.92–2.13)
13.50–45.00	46	104	1.35 (0.90–2.02)
Test for linear trend, *p*-value			0.11

Cumulative coal tar exposure (unit-years)			
0.39–4.29	43	105	1.31 (0.87–1.97)
4.30–9.42	32	100	0.98 (0.62–1.55)
9.43–16.88	30	105	0.97 (0.61–1.55)
16.89–196.48	54	100	1.60 (1.09–2.36)
Test for linear trend, *p*-value			0.07

Average exposure to coal tar (units)			
0.26–0.91	35	99	1.12 (0.72–1.76)
0.92–1.01	44	108	1.29 (0.85–1.97)
1.02–1.25	41	101	1.37 (0.89–2.11)
1.26–5.31	39	102	1.17 (0.77–1.79)
Test for linear trend, *p*-value			0.14

a Adjusted for set, country, age, tobacco pack-years.

b Referent group for all analyses shown in the table.
